# High-Frequency Oscillations in the Scalp Electroencephalogram: Mission Impossible without Computational Intelligence

**DOI:** 10.1155/2018/1638097

**Published:** 2018-08-07

**Authors:** Peter Höller, Eugen Trinka, Yvonne Höller

**Affiliations:** Department of Neurology, Christian Doppler Medical Centre and Centre for Cognitive Neuroscience, Spinal Cord Injury and Tissue Regeneration Center, Paracelsus Medical University, Salzburg, Austria

## Abstract

High-frequency oscillations (HFOs) in the electroencephalogram (EEG) are thought to be a promising marker for epileptogenicity. A number of automated detection algorithms have been developed for reliable analysis of invasively recorded HFOs. However, invasive recordings are not widely applicable since they bear risks and costs, and the harm of the surgical intervention of implantation needs to be weighted against the informational benefits of the invasive examination. In contrast, scalp EEG is widely available at low costs and does not bear any risks. However, the detection of HFOs on the scalp represents a challenge that was taken on so far mostly via visual detection. Visual detection of HFOs is, in turn, highly time-consuming and subjective. In this review, we discuss that automated detection algorithms for detection of HFOs on the scalp are highly warranted because the available algorithms were all developed for invasively recorded EEG and do not perform satisfactorily in scalp EEG because of the low signal-to-noise ratio and numerous artefacts as well as physiological activity that obscures the tiny phenomena in the high-frequency range.

## 1. Introduction

Epilepsy is one of the most frequent chronic neurological diseases affecting an estimated number of 6 million people of all age ranges in Europe and approximately 65 million people worldwide [1]. It significantly impacts not only a patient's health status but also quality of life factors, such as education, employment, social activity and integration, or mobility. The yearly treatment costs associated with epilepsy and its major psychiatric and somatic comorbidities add up to 15.5 billion in Europe or 2,000–11,500 per patient [2].

In spite of more than 15 antiepileptic drugs employing different modes of action to suppress or prevent seizures, with frequent mild-to-severe adverse effects, around ∼30% of patients, particularly suffering from focal epilepsy, remain resistant to drug treatment [1].

Epilepsy surgery is an important treatment option in view of this considerable percentage of pharmacorefractory cases. The intervention aims at removing the entire epileptogenic zone—a necessary condition in order to achieve postsurgical seizure freedom [3]. For successful surgical treatment, it is therefore critical to determine the epileptogenic zone as precisely as possible. There is, however, no diagnostic modality available today to unambiguously delineate the epileptogenic zone. It, thus, remains a theoretical construct that has to be estimated and assessed using a combination of diagnostic concepts based on a variety of parameters.

Throughout the last one and a half decades, increasing attention has been paid to fast and ultrafast electroencephalographic (EEG) oscillations as a measurable electrophysiological component of potentially pathological brain activity. Motivated by studies that consistently report a traceable correspondence between the postsurgical outcome and resection of brain tissue identified as a generator of high-frequency oscillations, frequency bands beyond the 80 Hz threshold as well as oscillations in the fast-gamma band (40–80 Hz) have been attracting considerable attention during the last decade throughout the epilepsy research community.

In fact, a number of recent studies have shown that the resection of areas that have been identified to generate (pathological) oscillations well beyond the 80 Hz boundary of conventional EEG recordings leads to favourable postsurgical results [4–6]. In particular, in patients with temporal lobe epilepsy, which is the most common type of drug-resistant epilepsy, the removal of brain tissue exposing the highest rates of high-frequency oscillations (HFOs) led to improved surgical outcomes [7], suggesting that HFOs may be a reliable and accurate (spatial) marker that should be taken into account in presurgical evaluation of patients [8]. It is important to note at this point that there is no precise specification of “HFO” commonly agreed upon. Reduced to a common denominator, it subsumes activities in frequency bands above 80 Hz, physiological as well as pathological, and of diverse origin, categorised into ripples, “*R*,” 80–250 Hz, and fast ripples, “FR,” 250–500 Hz [9, 10].

It was discovered that pathological interictal FR HFOs [11] delineate the seizure onset zone (SOZ) largely independent of and much more specific and accurate than epileptic spikes [12], as well as more reliably than an underlying, potentially noncongruent lesion [13]. Despite the still evolving understanding of the important role of high-frequency activity in the physiological context, such as memory consolidation, processing of sensory input, alertness, and arousal, today fast and ultrafast EEG activity is widely recognised as a promising marker of epileptogenicity [4, 8, 10].

## 2. Physiological HFOs

Studies have set out to examine the generator mechanisms of pathological HFOs in view of their meaning and relevance for the disease pattern [14]. Indeed, HFOs have been identified to reflect medication effects and disease activity and may suggest conclusions on disease severity [15, 16]. Although, compared to interictal spikes, HFOs were found to show a higher level of correlation with seizures and expose a more stable localisation, assessed ictally versus interictally [17], and their suitability as a predictive marker in the preictal period could not be confirmed [18].

It is possible that the small effect sizes for good postsurgical outcome when resecting HFO-generating areas [4] are due to the fact that physiological HFOs coexist with the pathological version and therefore obscure the clinical significance of these phenomena. It has been evidenced that fast oscillation also plays a major role in physiological states and processes, such as alertness or long-term memory consolidation [19, 20]. The coexistence of physiologic and pathologic HFOs was reported for kainic acid-treated rats and patients with epilepsy [21, 22]. It is difficult to determine whether HFOs represent pathologic (epileptic) or regular physiologic activity in epileptic patients [23–26]. For example, HFOs in the hippocamus are associated with memory consolidation in humans (e.g., [19, 20, 27]), but they were also associated with temporal lobe epilepsy in multiple studies [4].

Several studies focused on ways to reliably distinguish pathological HFOs from physiological HFOs based on the alertness level or sleep stages [28, 29], the relation to spikes and slow oscillations [30, 31], oscillatory background activity [32], duration and peak frequency [33], characteristics of connectivity and log power [34], or morphology [35]. However, none of these approaches claimed to reliably distinguish pathologic HFOs from physiologic HFOs—there always remains a significant overlap between the two phenomena. It remains an open question of whether computational intelligence could pave the way for a reliable distinction of pathological versus physiological HFOs. Nevertheless, for this purpose, it is inevitable to establish a ground truth or a valid strategy by which a model could be trained.

## 3. Scalp EEG and HD-EEG

A majority of studies on HFOs are based on intracranially recorded data. Due to important factors, such as cost or inherent risk of invasive procedures, the question whether HFOs are detectable using scalp EEG has been steadily moving into the focus of research.

Considering the plain number of studies, surface HFOs appear to have been comparatively disregarded for a long time. Difficulties in detecting genuine pathological fast oscillations—their recognition and distinction from, for example, muscle and filter artefacts is nontrivial—and, according to early studies, their rare measurable occurrence in only a small percentage of patients [12] might be reasons.

Similar to intracranial HFOs, surface fast oscillations are characterised by their low sensitivity but considerable specificity. Although research is still required to establish concepts to accurately distinguish genuine fast oscillations from artefacts in surface recordings [5], a positive correlation between fast oscillation rates and spike rates and a significantly higher frequency of occurrence inside than outside the SOZ confirm their relevance as a noninvasive marker of epileptogenicity. Studies by Melani et al. [36] and van Klink et al. [37] set scalp HFOs into context with measured spike rates and observe that consistent with findings based on intracranial data, scalp HFOs are less sensitive but more specific than epileptic spikes, with the highest HFO rates cooccurring with the highest IED rate.

Fast oscillations on the scalp have been identified in several studies [36, 38, 39], and the suitability of surface-recorded fast oscillations to demarcate seizure-generating tissue and to indicate its epileptogenic potential seems meanwhile undoubted [5, 39]. Despite considerable variations in the amplitude of the electric potential distribution on the scalp for any given extent of generator, caused by the local curvature of the cortex, thickness of the skull, and distance between the cortical surface and skull, it has been shown that, in general, the amplitude of background activity decreases with increasing frequency [40]. According to the authors' reasoning, it is consequently likely that small generators of high-frequency activity could produce scalp signals that could be detected with a reliability similar to other interictal epileptic discharges (IEDs). Nevertheless, the signal amplitude must be greater than noise, which might be possible as alongside with the drop of signal power, the noise level also diminishes [41].

More recent studies on fast oscillations in scalp recordings extended the frequency coverage below the common HFO band specifications and focused on the frequency band between 40 and 200 Hz; thus, the more general term “fast oscillations” is proposed (Table 1).

It has been a commonly accepted assumption that at least 6–10 cm^2^ of cortical tissue is required to generate epileptiform discharges that are measurable on the scalp and distinguishable from background activity, due to factors such as the conductive properties of the human skull or distance between the generator and measurement sites [51]. However, there might be likely cases for which the superposition of nonspatially contiguous generators could generate signals from smaller sources that result in a detectable potential on the scalp, as long as the potential is directly recorded from the right spot and as long as the signal-to-noise ratio is good enough [41]. Individual generators of high-frequency oscillations with a probable extent of 1 to 2 cm^2^ [40] were supposed to be too small to produce activity observable in scalp EEG recordings. It is possible that this regional restriction explains the fact that initially only a few studies set out to assess the detectability of HFOs using scalp EEG and that these studies reported to identify fast oscillations in a very small percentage of epileptic patients, only [12, 52].

Recent studies approach the question whether and via which mechanisms small generators can be seen on the scalp from different perspectives. A study recorded the EEG simultaneously from subdural grids and scalp EEG [45]. The study results suggest that HFOs originating from small patches of cortical tissue are in fact visible in the scalp EEG, provided that the signal-to-noise ratio is sufficiently large. Models that investigate the correlation of subdural voltage distributions and projections to the scalp lend this observation a theoretical foundation [40].

The studies [45] and [41] suggest that the rare occurrence of scalp HFOs may be due to spatial undersampling using conventional 10–20 setups and infer that a denser mesh of electrodes may be necessary to systematically study scalp HFOs. Apart from an extended coverage, high-density EEG systems (also referred to as “dense array” or “dEEG”) offer a finer-grained spatial resolution. The denser mesh of electrodes is considered an advantage in terms of sensitivity to high frequencies and their specific, rather local propagation patterns [45, 53], a conclusion which, however, was controversially discussed at the Second International Workshop on High-frequency Oscillations in Epilepsy, Freiburg, Germany, March 10–12, 2016.

Although a considerable number of publications exploit high-density EEG, with a majority of them focusing on source localisation (PubMed query results obtained in June 2017 reported 371 studies. Roughly one-third of them cover the localisation of signal sources), a targeted electronic literature search for HFO detection and high-density scalp EEG in PubMed (http://www.ncbi.nlm.nih.gov/pubmed; search string: (“hfo” or “high-frequency oscillations” or “high frequency oscillations” or “fast oscillations” or “ripples”) and (“HD-EEG” or “high-density” or “high density” or “dense-array” or “dense array” or “high-resolution”) and (“electroencephalography” or “eeg” or “electroencephalogram”)) performed in June 2017 revealed only a small set of nine studies, a single one of which was found to actually elaborate on the use of high-density scalp EEG for detecting high-frequency oscillations in epilepsy patients, although on a theoretical basis [45].

A number of recent studies set out to detect fast oscillations noninvasively in the MEG (e.g., [54–56]), with its typically dense mesh of sensors. However, MEG is associated with high costs, while long-term or bedside recordings are not possible. Thus, high-density scalp EEG remains an open and demanding field when it comes to analysing actual patient data. The assumed small size of cortical generators and, relative to invasive data, poor signal-to-noise ratio are frequently stated as reasons for unsatisfactory HFO analysis results in scalp recordings, like challenging visual identification as well as the set of widespread analytical detection strategies.

Up to now, investigations in scalp HFOs generally focus on ripples and so far failed in reliably detecting pathological oscillations in the frequency range above 200 Hz, which is more likely a consequence of the small-scale genesis and local propagation of high frequencies [53, 57], rather than caused by the frequency-dependent signal attenuation properties of the tissue [58]. Remarkably, a proof-of-concept study by Pizzo et al. [48] postulates that it may even be possible to detect extracranial fast ripples (>250 Hz) using subdermal electrodes. In a modelling study by von Ellenrieder et al. [40], simulations of signal sources of small extent and their electric potentials that make use of extremely detailed head models to analyse the noise patterns in different frequency bands support this assumption.

However, even for lower frequencies, it remains unclear whether the signal measurable on the scalp originates from the same structures and has the same generator mechanisms as data recorded invasively. Probably, phases of occasional synchronisation of larger regions are what becomes detectable on the surface [12].

To conclude, scalp HFOs would be an asset to clinical practice, but their detectability represents the largest challenge. Visual identification is prone to errors and extremely time-consuming, thus calling again for automation.

## 4. Automated HFO Detection

**Figure 1:**
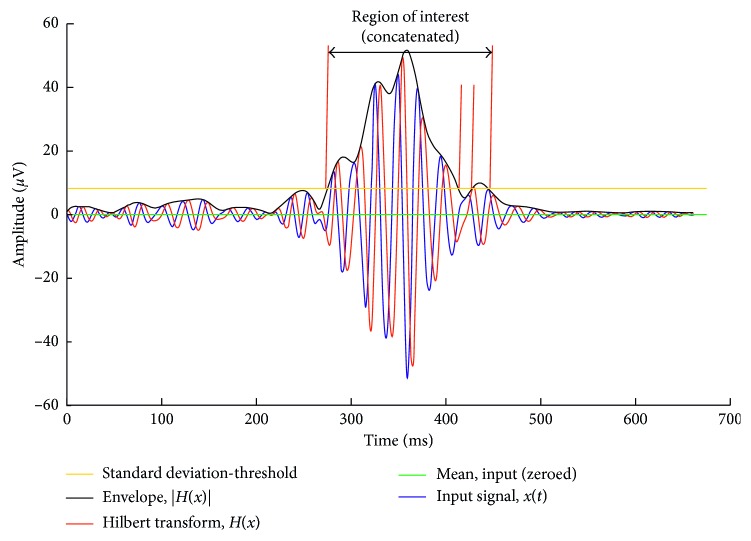
Definition of region of interest according to Burnos et al. [64]. The signal is high-pass filtered (finite impulse response, Blackman windowed sinc) with a cutoff frequency of 80 Hz. A Hilbert transform of the filtered signal (blue) yields a complex output with a 90-degree phase-shifted imaginary part (red). The absolute value of the Hilbert transform is used to generate the signal's envelope (black). The standard deviation of the signal's envelope is the baseline for deriving the threshold for delimiting regions of interest as a first step. As depicted in the figure, closely neighbouring regions are concatenated to form a single one.

Apart from the considerable amount of time it takes even for an expert neurologist to identify and categorise fast oscillations, the process is obviously prone to subjective perception and bias [38]. Automated diagnosis of epilepsy [59], automated detection of epileptic spikes [60], automated seizure detection [61], and even seizure prediction [62] were supported by advanced algorithms from digital signal processing, often alongside with artificial intelligence. These technical advances have also been introduced into HFO research and proposed concepts and algorithms for automated detection of HFOs [38, 63–80]. We replicated an example for a recent algorithm in Figure 1.

It was proposed to group detectors by their first processing stage, either by filtering the EEG to the HFO frequency band or by time-frequency analysis [41]. Another possible distinction would be in analytical algorithms, relying on certain heuristics such as the frequency band and the amplitude of the HFOs, and as a second group in the algorithms that involve artificial intelligence, relying on the features that are fed into the machine in order to build an adequate model for a given pattern. Machine-learning techniques vary to a large extent based on the way the model is trained. Support vector machines were suggested to be useful for HFO detection [81–83], but also linear discriminant analysis [84], as well as artificial neural networks [68, 85]. We expect that systematic research within this field could open up new perspectives beyond the analytical approach.

It is possible that the use of machine learning might be able to work through characteristics that are not so much dependent on the amplitude of the HFOs and the signal-to-noise ratio so that a broader use of HFO detection in routine scalp EEG could become possible one day. Especially convolutional neural networks raised the hope that one day we get a deeper understanding by posing our questions to a well-trained network for EEG analysis [86]. Nevertheless, deep-learning networks need extremely large amounts of data containing thousands of samples—this is a goal that can be achieved only in a concerted multicentric approach with large, shared databases. Finally, this speculation needs well-designed tests before entering the clinical arena in the form of a new clinical tool.

Most of the presented algorithms are based on spectral analyses of the data (using fast Fourier transform or wavelet techniques), either as part of an analytical approach or as a feature that is being used to train a model. It is understood, however, that these time-frequency models are not sufficient to distinguish pathological oscillations from physiological oscillations and that they are prone to errors when sharp transients cooccur with HFOs [81]. Supported by a steadily consolidating understanding of the generator mechanisms and propagation patterns of fast oscillations [26], this led to coherence measures, network and connectivity analyses, graph theoretical analysis, and related information theoretic approaches [14, 87–91]. The idea behind these advanced measures is that the characteristics of pathological HFOs differ from artefacts and physiological HFOs. For example, an artefact on the surface is detectable simultaneously over a large subset of the electrodes, while in contrast, scalp HFOs are detectable only over a small area. Measures of synchrony (i.e., connectivity) would therefore detect high synchrony over a large number of channels for artefacts, which could be a distinguishing feature. Another idea could be that the information content of pathological HFOs differs from that of physiological HFOs. Pathological tissue in the hippocampus is known to generate signals with low informational content, while signals recorded from healthy tissue are highly complex [92]. It could be investigated whether the pattern of HFO occurrences, that is, the inter-HFO time, distinguishes pathological HFOs from physiological HFOs. We would expect that pathological HFOs occur at a more regular pace.

A general problem of investigations in differentiation between pathological and physiological HFOs is the lack of a ground truth, which could be mitigated by using simulated data [82]. Moreover, heuristics were proposed to distinguish between these two categories. One possibility is to distinguish physiological HFOs from pathological HFOs based on their occurrence during cognitive effort or based on the location of occurrence. In brain regions such as the motor cortex, the visual cortex, and the hippocampus, we do expect physiological HFOs. Finally, the absolute ground truth would be relation of removal of HFO-generating tissue to good surgical outcome. If we could label the HFOs according to whether they were removed or not and according to whether the removal leads to a good outcome, we could relate this information to the categorisation of the HFOs of being pathological or not. Nevertheless, the obstacle here is that, in practice, the removal of tissue might include both, physiological and pathological HFOs. Moreover, while it is the current hope that the generation of HFOs may occur exactly within the epileptogenic zone, they could also rise from related areas. In the end, what could bring us a step forward is merging the knowledge among these approaches and generating a close-to-gold standard ground truth.

Although automated detection methods could also apply to surface recordings, most activities are based on invasively collected data. Only von Ellenrieder et al. [38] yielded remarkable results with automated HFO detection on scalp EEG, while most other publications involving scalp EEG relied on visual detection of HFOs. Most importantly, the effect sizes of resecting automatically detected HFOs versus resecting visually detected HFOs from invasive EEG in relation to surgical outcomes are comparable [4].

Nevertheless, currently we are lacking clinically approved tools for automated detection; that is, there are no software packages that are easy to use and validated by clinical trials so that their use within the standard clinical software by clinical staff is possible [41]. In contrast, there exist a large variety of open-source tools which are often MATLAB based and which where developed with research as the primary target application area, such as, for example, RIPPLELAB [93]. Other software such as MEEGIPS [94] was developed to integrate with the clinical workflow, but still, development of automated detection methods for a broad applicability in surface EEG is needed to render these tools more useful and to justify the long and costly process of clearance for clinical use.

Reliable automated detection is highly warranted in long-term recordings and becomes crucial for high-density recordings such as with magnetoencephalography or HD-EEG. But the performance of automated detection with high-density techniques is highly questionable.

## 5. Outlook

Even though principal methodological considerations on automation-supported HFO detection likewise apply to invasive and scalp recordings, again, almost all published activities are based on invasively collected data. The potential benefits due to the noninvasiveness of scalp EEG—lower risk, lower costs, and the possibility to include larger patient populations with different types of epilepsies as well as to conduct longitudinal studies—are unquestioned.

Whether the use of high-density EEG instead of conventional (10–20) systems as utilised in related recent research in combination with specifically adapted computer-supported detection mechanisms that take into account also the differentiation between pathological and physiological HFOs can be considered a promising step ahead which may broaden the use of scalp HFOs as biomarkers remains to be assessed. Particular challenges for HD-EEG-tailored detection algorithms must be considered: the large number of channels in high-density EEG recordings, the comparatively low signal-to-noise ratio, and a variety of artefacts that do not occur to the same extent in invasive recordings.

We encourage the implementation of the following approaches into research programs and funding of the respective efforts, in order to propel automated HFO detection into clinical routine:

- Clinical and technical research is often done by separate teams, and it would be highly beneficial if the precious data being collected at large clinics or within multicentric efforts could be the base for high-level data analysis of technical experts. For example, deep-learning networks need thousands of datasets but can draw amazing conclusions based on the information contained in these giant volumes of signal data. Therefore, we suggest that databases with human-annotated data should be published. Such databases are already available for seizure prediction and detection, allowing contests between expert teams [62, 95, 96]. The necessary protection of privacy and security should be supported by dedicated services and clear guidelines.
- Simulated data can help to explain phenomena and to explore the limits of the algorithms [97]. Simulation studies could serve as a standard test set, just as open-access or shared datasets do.
- Since today we do not know how to distinguish pathological HFOs from physiological HFOs, we cannot expect that even the best model could solve this problem for us. We need some heuristics by which we can feed some initialising information into a machine, such as the relation between HFO removal and surgical outcome or the functional relevance of HFOs due to their activation during tasks or due to their localisation. Machines which are trained based on the merged information from these three heuristics could come closer to the answer we aim to obtain.
- Several recent publications implemented artificial intelligence into automated HFO detection algorithms [68, 81–85]. We assume that new developments, for example, the use of convolutional deep-learning networks [86], in combination with the necessary large databases could identify new perspectives on the detection of HFOs.

## Figures and Tables

**Table 1:** Epilepsy-related HFOs in conventional surface EEG and MEG.

Reference	Frequency range	Detection	Context
Kubota et al. [42]	300–900 Hz	Visual	MEG benign rolandic epilepsy
Kobayashi et al. [43]	93.8–152.3 Hz	Visual	Idiopathic partial epilepsy
Andrade-Valenca et al. [12]	40–200 Hz	Visual	Comparison to spikes
von Ellenrieder et al. [38]	40–200 Hz	Auto	Autodetection
Iwatani et al. [44]	30–150 Hz	Visual	Spasms in West syndrome
Melani et al. [36]	40–200 Hz	Visual	Comparison to spikes
Zelmann et al. [45]	80–300 Hz	Auto/visual	Intracranial versus scalp HFOs
Miao et al. [46]	80–500 Hz	TF + visual	Absence epilepsy
Chaitanya et al. [47]	80–250 Hz	Visual	Absence epilepsy
Pizzo et al. [48]	>250 Hz	Visual	Scalp fast ripples
van Klink et al. [37]	80–250 Hz	Visual	Scalp ripples and spikes
van Klink et al. [49]	>80 Hz	Visual	MEG virtual sensors
Schwimmbeck et al. [50]	80–250 Hz	Auto/visual	Intracranial versus HD-EEG

TF: time-frequency analysis; auto: automated algorithmic detection.
